# Influence of Age at Diagnosis and Time-Dependent Risk Factors on the Development of Diabetic Retinopathy in Patients with Type 1 Diabetes

**DOI:** 10.1155/2016/9898309

**Published:** 2016-04-26

**Authors:** Luis Forga, María José Goñi, Berta Ibáñez, Koldo Cambra, Marta García-Mouriz, Ana Iriarte

**Affiliations:** ^1^Department of Endocrinology and Nutrition, Complejo Hospitalario de Navarra, Instituto de Investigación Sanitaria de Navarra (IdiSNA), Calle Irunlarrea 3, Pamplona, 31008 Navarra, Spain; ^2^Navarrabiomed, Fundación Miguel Servet, Red de Investigación en Servicios Sanitarios en Enfermedades Crónicas (REDISSEC), Calle Irunlarrea 3, Pamplona, 31008 Navarra, Spain

## Abstract

*Aim*. To determine the influence of age at onset of type 1 diabetes and of traditional vascular risk factors on the development of diabetic retinopathy, in a cohort of patients who have been followed up after onset.* Methods*. Observational, retrospective study. The cohort consists of 989 patients who were followed up after diagnosis for a mean of 10.1 (SD: 6.8) years. The influence of age at diagnosis, glycemic control, duration of diabetes, sex, blood pressure, lipids, BMI, and smoking is analyzed using Cox univariate and multivariate models with fixed and time-dependent variables.* Results*. 135 patients (13.7%) developed diabetic retinopathy. The cumulative incidence was 0.7, 5.9, and 21.8% at 5-, 10-, and 15-year follow-up, respectively. Compared to the group with onset at age <10 years, the risk of retinopathy increased 2.5-, 3-, 3.3-, and 3.7-fold in the groups with onset at 10–14, 15–29, 30–44, and >44 years, respectively. During follow-up we also observed an association between diabetic retinopathy and HbA1c levels, HDL-cholesterol, and diastolic blood pressure.* Conclusion*. The rate of diabetic retinopathy is higher in patients who were older at type 1 diabetes diagnosis. In addition, we confirmed the influence of glycemic control, HDL-cholesterol, and diastolic blood pressure on the occurrence of retinopathy.

## 1. Introduction

Diabetic retinopathy (DR) remains a common complication in people with type 1 diabetes (T1D). 39, 55, and 84% of T1D patients will develop DR after 10, 20, and 40 years of evolution, respectively [1–3]. The duration of diabetes and the glycemic control are the risk factors that are most closely related with all forms of DR. Other factors such as male gender, hypertension, high body mass index (BMI), nephropathy, dyslipidemia, smoking, and genetic factors appear to influence the onset or progression of DR, although their role is controversial [1, 3–5].

The influence of age at T1D diagnosis on the occurrence of microvascular complications like DR is currently a subject of active debate. Some studies have reported that the prepubertal stage, especially the first 5 years of life, might protect from the occurrence of DR [6–8]. However, there are authors that point out that such protection disappears as the disease progresses [2, 5], while others have never observed such an effect [9, 10].

Puberty has a negative influence on the appearance of DR, which is due to the combination of hormonal changes and the poorer control that often accompanies this stage of life [11]. When T1D onset is after age 15, the literature on the effect of age on DR development is limited and rather confusing. According to Hammes et al. [3], patients aged over 15 at onset have the lowest protection against advanced DR, whereas Hietala et al. [4] stated that the risk of proliferative DR (PDR) is higher when T1D onset is between 5 and 14 than when it is between 15 and 40. Finally, Kullberg et al. [12] reported that the prevalence of DR increases in patients aged 15 to 19 at onset but decreases at onset ages between 30 and 35.

In this study we take advantage of the cohort of patients included in the Type 1 Diabetes Registry of Navarra to estimate the risk of DR development according to age at onset and duration of T1D, smoking, blood pressure (BP), BMI, glycemic control as estimated by HbA1c levels, and lipid profile.

## 2. Methods

This is an observational retrospective follow-up study. The subjects of the study are all included in the T1D Registry of Navarra: patients with onset of T1D from January 1990, who were followed up and treated in the “Complejo Hospitalario de Navarra” until July 2013. The cohort included 989 patients. The study protocols were approved by the regional Ethical Review Board of Navarra.

T1D was diagnosed according to clinical criteria as recommended by the World Health Organization [13]. The clinical diagnostic criteria are those previously validated by Molbak et al. [14]. In all cases, we also measured anti-GAD and anti-IA2 antibodies. According to the medical protocol followed, all patients had at least one scheduled outpatient appointment per year. The patients' data needed for the study were obtained from the electronic health records of the Navarra Health Service. We gathered information about age and sex at onset for all patients. At the screening visit and for every visit through follow-up, we included weight, height, systolic BP (SBP), diastolic BP (DBP), smoking status, and analytical data such as lipid profile and HbA1c. When patients had more than one determination of these covariates in a year, we computed the arithmetic mean in the case of continuous variables, while for the categorical ones we chose the value that lasted longest in that year.

In all follow-up visits, smoking habits were ascertained and patients were categorized as nonsmokers, ex-smokers, or smokers. A nonsmoker was defined as someone who had smoked fewer than 100 cigarettes in their lifetime; an ex-smoker was someone who had smoked more than that amount but had quit smoking at least one year before the analysis of data was performed; finally, a smoker was someone who had not quit smoking or had quit within the last year.

BP was measured once, after a rest of at least ten minutes.

BMI was calculated using the formula: weight (in kilograms) divided by height (in meters) squared.

Screening and grading for the presence or absence of DR were performed by trained ophthalmologists using fundoscopy in mydriasis at least once every two years, starting five years after diagnosis. In our hospital, T1D patients are always explored by an ophthalmologist. Retinal examination by binocular biomicroscopy and a 78/90 D lens was recorded in a standardized format in the electronic health record of the Navarra Health Service. In patients under 12 years, the standard exploration consisted of indirect ophthalmoscopy with a 20/28 D lens. Retinopathy was graded according to a 5-degree severity scale based on the American Academy of Ophthalmology's simplified classification [15]. However, since we had rather few cases of PDR, all grades were grouped together for statistical purposes.

From 1990 to 1997, HbA1c was measured using various techniques (Abbott IMX, Ciba Corning Glycomet, Merck, and Menarini HPLC), but after 1997, HbA1c was determined in all patients with high-performance liquid chromatography (HPLC; Adams A1c HA, Menarini Diagnostics, Florence, Italy; reference range: 4.1–6.2%). In 2005, the Hospital Complex obtained level II laboratory certification of traceability from the Diabetes Control and Complications Trial (DCCT) reference method through the National Glycohemoglobin Standardization Program. Previous HbA1c determinations had also been standardized to the DCCT reference range [16].

HDL-cholesterol and triglycerides (TG) were measured by GPO-PAP (Roche Diagnostics). LDL-cholesterol was calculated by the Friedewald equation.

*Statistical Analysis*. Characteristics of the patients at onset of the disease were summarized using frequency and percentages for categorical variables and mean and standard deviations (SD) for continuous ones. The cumulative incidence of retinopathy was estimated and graphed for the whole sample and also divided by age groups. 95% confidence intervals based on the cumulative hazard were estimated for 5, 10, and 15 years after onset. Data were right-censored when no retinopathy event occurred during follow-up or due to loss to follow-up or death.

In order to assess the effect of the different variables on retinopathy, firstly, univariate Cox-proportional hazards regression models were fitted. We assessed the effect of covariates at onset as fixed effects and complemented the analyses with a dynamic approach that includes the covariates as time-dependent variables, updating the values along the follow-up. The proportionality assumption implicit in the Cox models was assessed using weighted residuals and when violated, an interaction term with time was evaluated. The possible modifying effect of age group was evaluated and models were adjusted by age group when appropriate. Secondly, a multivariate regression model was fitted with the covariates that had turned out to be significant in the previous step.

All analyses were performed using the R statistical package, version 3.1.1.

## 3. Results

989 patients with T1D were followed up from onset, with mean (SD) follow-up of 10.1 (6.8) years. Of them, 292 (29.5%) had the onset in childhood (under 15 years) and 579 (58%) were men. At onset, 8 (0.8%) had lipid lowering treatment and 12 (1.2%) antihypertensive treatment, figures that increased to 143 (14.5%) and 103 (10.4%), respectively, at follow-up. Antihyperlipidaemic and antihypertensive treatment were more frequent in patients with than without retinopathy (20.7% versus 13.5% and 21.5% versus 8.7%, resp.).

Demographic and clinical characteristics are shown in Table 1.

At follow-up, 135 patients developed retinopathy (13.7%), 121 of whom had nonproliferative retinopathy (NPDR) and 14 PDR. All patients with PDR had been previously diagnosed with NPDR. Given the low number of patients with PDR, statistical results are focused on total retinopathy. Nevertheless, it deserves to be mentioned that, at onset, patients that develop PDR have similar HbA1c mean values compared to the rest of the patients (10.9 (4.1) versus 11.0 (2.5)), but at follow-up, patients that developed PDR had HbA1c mean values of 9.39 (2.01), whereas those that developed NPDR had 8.30 (1.51), and those with no retinopathy had 7.74 (1.33).

**Figure 1:**
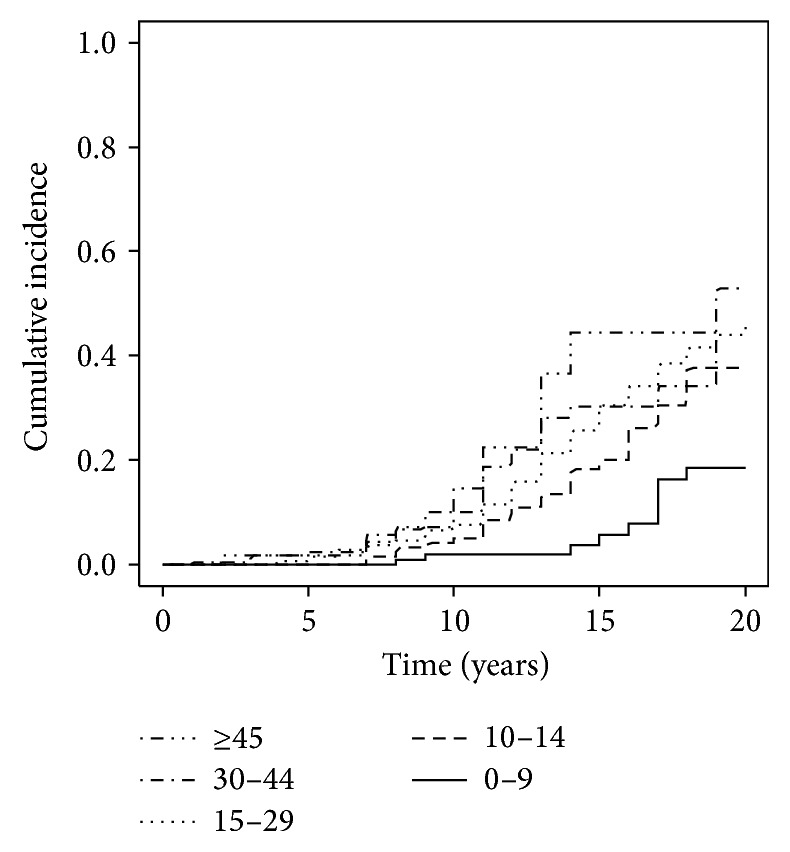
Cumulative incidence of retinopathy by age group.

As expected, the cumulative incidence increased over time during the course of diabetes. It was very low during the first 5 years after onset, but it undoubtedly increased after 10 and, especially, 15 years. We observed that the rate of retinopathy was higher in patients that were older at diagnosis. The highest increase was observed in the group whose onset was at age ≥45 years. Notably, after 15 years of follow-up its cumulative incidence was 12 times higher than that observed in the group of patients whose onset was at age <10 years (Table 2 and Figure 1).

In the univariate analysis, male gender, smoking, SBP, and HDL-cholesterol at onset were significantly associated with the risk of DR throughout follow-up. However, the association of HbA1c with DR development was only marginally significant (*p* = 0.079). Remarkably, when taking the patients who were younger than 10 at onset as the reference group, the risk increased according to age at onset (Table 3). When an additional univariate analysis was performed for each variable according to its evolution along the follow-up period, a significant association with DR development was again observed for smoking, SBP, and HDL-cholesterol, with HRs (95% CI) similar to the former ones. Unlike what happened when the analysis was performed using values at onset, DBP, triglycerides, BMI, and HbA1c were now significantly associated with DR. Finally, the association between LDL-cholesterol and DR was slightly above the limit of statistical significance [1.06 (1.00, 1.13), HR (95% CI), *p* = 0.052] (Table 4).

The multivariate analysis confirmed the trend observed in the univariate one and the risk of developing DR increased according to age at onset, albeit less markedly (Table 5). In any case, when focusing on pediatric age, children with onset at the peripubertal period exhibited a significantly higher risk when compared with those <10, the HR increasing more than 2-fold. In adult patients, the risk of DR development increased more than three times with respect to children <10, and, notably, it was almost 4 times higher in the group of patients ≥45 at onset [HR 3.78 (95% CI: 1.37–10.41)]. The multivariate approach confirmed that, among the time-dependent variables, DR development along follow-up was also significantly influenced by DBP, HDL, and HbA1c, the latter when values were above 9% (Table 5).

## 4. Discussion

The rate of DR increases with the age of diagnosis. Taking advantage of the follow-up of the patients included in the T1D Registry of Navarra, we have confirmed the influence of some controversial risk factors on the occurrence of DR. Remarkably, we describe for the first time that the risk of DR 15 years after diagnosis increases with increasing age of onset, being highest in those with onset at age ≥45.

HbA1c is the factor exhibiting the highest impact on the development of DR [3]. In the Wisconsin study [17], the HR (95% CI) to develop DR for patients with HbA1c from 9.5 to 10.5, compared with patients with HbA1c levels <9.5%, is 1.72 (1.34, 2.21). However, it rises to 2.41 (1.91, 3.06) when HbA1c ranges from 10.6 to 12% and is even higher, 3.65 (2.87, 4.65), for HbA1c values >12%. We cannot compare these results with ours, because our highest quartile of HbA1c is lower than the lowest of theirs, but both studies are consistent in showing the relationship between poor metabolic control, as measured by glycated hemoglobin, and DR development and progression. This relationship has been shown in several publications based on national or regional T1D records, some of them recently [1, 18].

T1D duration is the other factor clearly related to the onset and progression of DR [3, 17], and in our series this association was also observed.

Regarding the factors most frequently discussed, our results match those published on the risk of DR [1, 3, 17], which is higher in men than in women, although this difference disappears when the effect of confounding variables is prevented by the multivariate analysis, suggesting that the risk is due to factors other than gender.

The relationship between BP and DR has been generally accepted [1], but results have not always coincided. Published data are influenced by the type of analysis and are divergent if analysis of BP is performed at baseline or throughout follow-up, if BP is stratified by ranges or by 10 mmHg increments, or if the comparison is only between hypertensive and normotensive patients [3, 17]. Different results have also been reported depending on how DR is evaluated: as a whole, or taking NPDR and PDR cases separately, or even if the progression from one to the other is analyzed. Our results largely match those of the Wisconsin study [17], although the latter treats NPDR and PDR as different entities and we have analyzed them in a single group since there were only 14 patients with PDR in the T1D Registry of Navarre.

The association between smoking and DR is controversial: Hammes et al. [3] found a significant relationship, while other authors disagree [19]. Our results are in accordance with those of the Linkoping Diabetes Complications Study [20] in that although smoking is associated with the risk of DR in the univariate analysis, there is no relationship between both variables after adjusting for confounding factors in the multivariate analysis. Therefore, there must be other factors closely associated with smoking to explain this recurrent finding. The fact that smoking is linked with a worse glycemic control may be responsible for such an observation [21].

Dyslipidemia, especially increased triglyceride and decreased HDL-cholesterol levels and, to a lesser extent, the increase in total cholesterol and LDL-cholesterol, is a risk factor, albeit weak, for the occurrence of DR, especially in its severe forms [22, 23]. Thus, additional factors (genetic, inflammatory, and metabolic) might be necessary for lipids to induce such effect. In any case, there is experimental evidence that increased and biochemically altered plasma lipids lead to cytotoxicity in retinal capillary cells [3]. In our study the findings on LDL- and HDL-cholesterol and triglycerides (Tables 3, 4, and 5) are consistent with the data published on a Finnish population [22].

The occurrence of DR (PDR plus NDRP) has been related to high BMI in the DIS study in Sweden [1], but, in Finland, Hietala et al. [4] have not found any influence of BMI on the risk of PDR. Our results, in the multivariate analysis, differ from those obtained in Sweden, perhaps due to a smaller number of events in our series (135 versus 247 patients).

The influence of age of diagnosis on the onset and progression of DR has been the subject of numerous publications; most of them focused on children and the probable protective effect of the prepubertal period. Ours is the first study to include patients who have been diagnosed with T1D over the age of 40.

In our series, patients within the pubertal period, that is, children aged 10–14 years, exhibit a risk of developing DR which is 2.5 times higher than the risk associated with children who are aged 0–9 years. These data are consistent with the results of most authors [5–8], especially those of Olsen et al. [10], but differ from those obtained by Holl et al. [9] and by the Wisconsin study [24].

There is a controversy about the effect of hyperglycemia on the occurrence of microvascular complications before puberty. Discrepancies may be due to different interpretations about what is the exact range of age encompassed by the term puberty, which have led to heterogeneous ways of grouping patients aged <15 years. There is a real need to gain knowledge on this topic to take advantage of the age at diagnosis to help in deciding the extent of the aggressiveness of insulin treatment [2].

During puberty, glycemic control is worsened and the risk of microangiopathy development increases [25], which is partly due to insulin resistance. The insulin sensitivity in the middle of puberty is reduced by 30–35% compared to the late stage of puberty, prepubertal childhood, or adulthood. This seems to be mainly due to the effects of the growth hormone (GH) and the insulin growth factor-1 (IGF-1) [11]. The actions of GH are mediated by IGF-1, and the levels of both molecules have been correlated with the thickness of the capillary basement membrane as well as with diabetic angiopathy. The increased GH and IGF-1 during puberty are related with the increased gonadal steroids. Furthermore, the increased levels of sex hormones are directly related to vascular structural abnormalities associated with diabetes complications. This is due to the ability of such molecules to increase polyol metabolism in the basement membrane, as has been demonstrated in animal models [26]. There is an association between capillary basement membrane thickening and the duration of T1D in the postpubertal period. In fact, diabetes control markers, such as HbA1c and fasting glucose, are positively correlated with thickness in postpubertal but not in prepubertal patients, thus suggesting an interaction between diabetes control and puberty [8]. Psychosocial factors associated with adolescence may also contribute to a poorer glycemic control during this phase and thus to its subsequent undesirable effect on morbidity in patients with T1D [9].

Our patients aged 15 to 29 years have a risk of DR which is 3-fold the risk exhibited by children <10. Not surprisingly, these results are again in agreement with those obtained by Olsen et al. from the Danish Study Group of Diabetes in Childhood [10] and in disagreement with those recorded in the Wisconsin study [17]. In our series, the risk continues to increase with age, so that it is even higher for patients >30 and, especially, almost 4-fold, for patients ≥45. Hammes et al. [3] also found an increased risk for PDR in patients diagnosed with T1D and aged 15 to 40 years. By contrast, Kullberg et al. [12], studying patients aged up to 36 at onset and also gathering all forms of retinopathy, observed a decrease in the prevalence of DR in patients older than 30. In the Finnish records [4], the risk of PDR was lower in patients who were aged 15 to 40 at onset compared to those whose onset was at puberty. The beta cells are deemed to be best preserved when diabetes onset is in adulthood. As a consequence, the glycemic control would be easier when development of T1D was at an age long after puberty, and this would explain the lower risk of DR found in these patients. Our group had previously published [27] that, in the patients included in the T1D Register of Navarra, a relationship between the age of onset and glycemic control also exists. However, in our cohort the worst glycemic control at follow-up was observed in the group of oldest patients at T1D onset. Thus, from this point of view our present findings about age at onset and risk of DR are consistent with our previous observations.

The current study is not exempt from limitations. The main one is that the number of patients who developed PDR was rather small in our cohort. For this reason, we were not able to analyze separately NPDR and PDR; that is, we do not have information about whether or not there are risk factors that influence differently the progression to one entity or to the other. We are confident that longer follow-up, lasting over 20 years, will allow us to obtain more information in the future.

We also consider that there are strengths in this work. Among them, we want to remark that our cohort includes a significant number of patients who were followed up from diagnosis. In fact, the onset of disease of each patient is the time of his/her inclusion in the study, which allows the analyses of incidence at different times, that is, 5, 10, or 15 years, to be more accurate than if the onset and baseline had not matched. Furthermore, we consider that the risk of bias that could have led to inaccurate results is rather low since the follow-up of all patients took place at the same hospital, and the screening and grading for the presence of DR were always performed by the same ophthalmologists, who followed similar clinical criteria throughout the study.

In sum, the analysis of the T1D Registry of Navarra shows, for the first time, that there is a relationship between the age at T1D diagnosis and the prevalence of DR throughout life and confirms the influence of T1D duration and glycemic control on the appearance of DR. Our results also support the view that BP and lipid profile have an effect on DR.

## Figures and Tables

**Table 1:** Demographic and clinical characteristics at onset of our population of T1D patients.

Variable		Total
Categorical variables		*n* (%)

Sex		
Men		579 (58%)
Women		410 (42%)
Age group (years)		
0–9		190 (19%)
10–14		192 (19%)
15–29		334 (34%)
30–44		204 (21%)
≥45		69 (7%)
Smoking		
No		613 (68%)
Ex-smoker		42 (5%)
Yes		249 (27%)
Antihypertensive treatment		
No		981 (99.2%)
Yes		8 (0.8%)
Lipid lowering treatment		
No		977 (98.8%)
Yes		12 (1.2%)

Continuous variables	*n* = 989	Mean (SD)

Years of follow-up	989	10.1 (6.8)
SBP (mmHg)	816	115.7 (14.8)
DBP (mmHg)	816	70.1 (11.4)
LDL (mg/dL)	783	116.5 (40.3)
HDL (mg/dL)	804	48.7 (15.2)
Triglycerides (mg/dL)	840	119.9 (150.9)
BMI	821	20.2 (4.4)
HbA1c (%)	793	11.0 (2.5)

SBP, systolic blood pressure; DBP, diastolic blood pressure; LDL, low density lipoproteins; HDL, high density lipoproteins; BMI, body mass index; HbA1c, glycated hemoglobin.

**Table 2:** Cumulative incidence (IC 95%) after 5, 10, and 15 years since onset.

Cumulative incidence of DR			
	Time since onset		
	5 years (CI)	10 years (CI)	15 years (CI)
Overall T1D population	0.7 (0.1, 1.3)	5.9 (4.0, 7.7)	21.8 (17.7, 25.7)

T1D groups by age at onset			
0–9 years	0	2.0 (0, 4.8)	3.7 (0, 7.9)
10–14 years	0	4.2 (0.5, 7.7)	18.3 (9.4, 26.3)
15–29 years	0.8 (0, 1.8)	6.5 (3.3, 9.7)	25.8 (18.8, 32.2)
30–44 years	1.7 (0, 3.6)	10.0 (4.3, 15.3)	30.3 (18.1, 40.7)
≥45 years	1.8 (0, 5.1)	7.3 (0, 15.1)	44.5 (14.7, 63.9)

**Table 3:** Univariate analysis to analyze the association between age, gender, and other risk factors at T1D onset with the development of DR during the subsequent follow-up period.

Variable	HR (CI 95%)	*p* value
Sex		
Male	Reference	
Female	0.56 (0.39, 0.80)	0.001
Age (years)		
<10	Reference	
10–14	2.58 (1.24, 5.37)	
15–29	3.64 (1.87, 7.10)	<0.001
30–44	4.23 (2.04, 8.76)	
≥45	5.32 (2.21, 12.84)	
Smoking		
No/ex-smoker	Reference	
Smoker	1.68 (1.16, 2.44)	0.007
BMI	1.12 (0.94, 1.33)	0.274
SBP (per 10 mmHg)	1.15 (1.02, 1.31)	0.034
DBP (per 10 mmHg)	1.12 (0.94, 1.33)	0.203
HDL (per 10 mg/dL)	0.75 (0.64, 0.88)	<0.001
LDL (per 10 mg/dL)	1.01 (0.97, 1.06)	0.606
Triglycerides (per 10 mg/dL)	1.01 (1.00, 1.02)	0.174
HbA1c (per 1%)	1.09 (0.99, 1.19)	0.079

The HR given for quantitative variables refers to increments of 10 units for all covariables except for HbA1c, for which it is referred to increments of 1% points. BMI, body mass index; SBP, systolic blood pressure; DBP, diastolic blood pressure; LDL, low density lipoproteins; HDL, high density lipoproteins; HbA1c, glycated hemoglobin.

**Table 4:** Univariate analysis to analyze the association between time-dependent variables along the follow-up and the development of DR during the same period.

Variable	HR (CI 95%)	*p* value
Smoking		
No/ex-smoker	Reference	
Smoker	1.75 (1.24, 2.47)	0.001
SBP (per 10 mmHg)	1.28 (1.14, 1.45)	<0.001
DBP (per 10 mmHg)	1.75 (1.44, 2.12)	0.001
HDL (per 10 mg/dL)	0.78 (0.69, 0.88)	<0.001
LDL (per 10 mg/dL)	1.06 (1.00, 1.13)	0.052
Triglycerides (per 10 mg/dL)	1.04 (1.02, 1.06)	<0.001
BMI	1.10 (1.05, 1.15)	<0.001
HbA1c (per 1%)	1.22 (1.08, 1.37)	0.001

SBP, systolic blood pressure; DBP, diastolic blood pressure; LDL, low density lipoproteins; HDL, high density lipoproteins; BMI, body mass index; HbA1c, glycated hemoglobin.

**Table 5:** Multivariate analysis to analyze the association of the development of DR with age at T1D onset and with DBP, HDL, and HbA1c along the follow-up.

Variable	HR (CI 95%)	*p* value
Age at onset (years)		
<10	Reference	
10–14	2.57 (1.15, 5.76)	
15–29	3.04 (1.44, 6.47)	0.012
30–44	3.35 (1.49, 7.56)	
≥45	3.78 (1.37, 10.41)	
DBP (per 10 mmHg)	1.55 (1.26, 1.91)	<0.001
HDL (per 10 mg/dL)	0.77 (0.68, 0.88)	<0.001
HbA1c		
≤7%	Reference	
7-8%	1.34 (0.72, 2.46)	
8-9%	1.69 (0.92, 3.11)	0.009
>9%	2.56 (1.38, 4.75)	

DBP, diastolic blood pressure; HDL, high density lipoproteins; HbA1c, glycated hemoglobin.
